# A Survey on ML Techniques for Multi-Platform Malware Detection: Securing PC, Mobile Devices, IoT, and Cloud Environments

**DOI:** 10.3390/s25041153

**Published:** 2025-02-13

**Authors:** Jannatul Ferdous, Rafiqul Islam, Arash Mahboubi, Md Zahidul Islam

**Affiliations:** School of Computing, Mathematics and Engineering, Charles Sturt University, Albury, NSW 2640, Australia; mislam@csu.edu.au (R.I.); amahboubi@csu.edu.au (A.M.); zislam@csu.edu.au (M.Z.I.)

**Keywords:** machine learning, malware detection, multi-platform malware, malware analysis, PC malware, mobile malware, IoT malware, cloud-based malware detection

## Abstract

Malware has emerged as a significant threat to end-users, businesses, and governments, resulting in financial losses of billions of dollars. Cybercriminals have found malware to be a lucrative business because of its evolving capabilities and ability to target diverse platforms such as PCs, mobile devices, IoT, and cloud platforms. While previous studies have explored single platform-based malware detection, no existing research has comprehensively reviewed malware detection across diverse platforms using machine learning (ML) techniques. With the rise of malware on PC or laptop devices, mobile devices and IoT systems are now being targeted, posing a significant threat to cloud environments. Therefore, a platform-based understanding of malware detection and defense mechanisms is essential for countering this evolving threat. To fill this gap and motivate further research, we present an extensive review of malware detection using ML techniques with respect to PCs, mobile devices, IoT, and cloud platforms. This paper begins with an overview of malware, including its definition, prominent types, analysis, and features. It presents a comprehensive review of machine learning-based malware detection from the recent literature, including journal articles, conference proceedings, and online resources published since 2017. This study also offers insights into the current challenges and outlines future directions for developing adaptable cross-platform malware detection techniques. This study is crucial for understanding the evolving threat landscape and for developing robust detection strategies.

## 1. Introduction

In recent years, malware has evolved into one of the most pervasive cybersecurity threats, capable of targeting not only traditional systems such as PCs, but also mobile devices, IoT, and cloud platforms. Malware is becoming increasingly complex and varied by employing methods such as code obfuscation, encryption, polymorphism, and metamorphism to avoid detection [1]. The increasing sophistication of malware and its capability to bypass conventional security measures have caused significant financial, operational, and reputational damage to individuals, businesses, and governments. As technology becomes increasingly integrated across platforms, cybercriminals can simultaneously exploit multiple systems. Therefore, a thorough investigation of multi-platform malware detection is not only timely but crucial for ensuring cybersecurity resilience.

In the context of cybersecurity, malware refers to malicious software that is intentionally designed to disrupt, damage, or gain unauthorized access to computer systems. In contrast, multi-platform malware is malware capable of infecting and spreading across various types of platforms, often simultaneously. Malware can be categorized into several forms depending on its purpose and information-sharing system, such as ransomware, spyware, adware, rootkits, worms, horses, botnets, trojans, and viruses. Machine learning (ML), in this study, refers to computational techniques that allow systems to learn from data and improve their performance over time without being explicitly programmed. The application of ML for malware detection has shown significant potential for automating threat identification and reducing detection latency, especially in environments with high complexity and variability.

**Figure 1:**
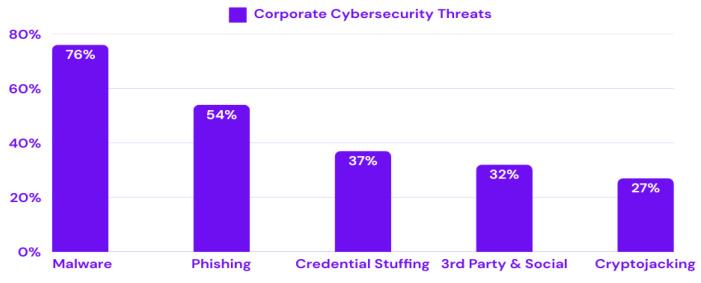
Types of attacks experienced by companies (published September 2024 by CISCO) [2].

The increasing prevalence of malware is evidenced by the increasing number of global cyberattacks. According to the 2024 Cisco Cybersecurity Readiness Index [2], 76% of firms experience malware attacks, as shown in Figure 1. Astra’s Malware Statistics 2024 reports that 560,000 new malware pieces are detected daily, adding to over 1 billion existing programs. This large volume of malware thwarts organizational security, often resulting in ransomware attacks [3]. The scale and impact of ransomware attacks are expected to increase significantly in the future. Cybersecurity Ventures predicts that victims could pay approximately USD 265 billion annually by 2031, with costs increasing by 30% each year [4]. Malware targeting Linux systems has also increased, with a 35% increase in infections and the emergence of new malware families impacting Linux-based platforms [5]. Furthermore, 2023 marked a pivotal moment for IoT security threats. A report from Zscaler ThreatLabz in October 2023 showed a 400% increase in IoT malware attacks compared to the previous year [6]. Overall, the global proliferation of mobile devices, IoT systems, and cloud computing has expanded the attack surface, providing cybercriminals with new vectors for deploying malware. Hence, new challenges have arisen for malware detection. Traditional malware detection methods tailored to specific platforms, such as PCs or mobile devices, are insufficient to counter these new threats. This underscores the necessity of adopting a unified, multi-platform approach to malware detection that can provide holistic defense strategies.

In response to this evolving threat landscape, numerous studies have focused on machine learning (ML) owing to its ability to handle modern threat complexity. Traditional malware detection approaches, such as signature-based and heuristic methods, have proven inadequate for sophisticated and polymorphic malware, especially in dynamic, multi-platform environments. Thus, the adoption of advanced detection techniques, including behavior-based and ML-driven approaches, has become essential in modern cybersecurity defense. In this context, neural network models, including convolutional neural networks (CNNs) and recurrent neural networks (RNNs), have been widely adopted for processing data in malware detection. CNNs excel in analyzing binary files and images, whereas RNNs are effective for sequential data such as system call sequences [7]. Additionally, recent advancements in large language models (LLMs) [8], such as Generative Pre-trained Transformer (GPT) and Bidirectional Encoder Representations from Transformers (BERT), have opened new avenues for malware detection by analyzing textual features like audit logs, threat intelligence reports, and API call sequences. These models capture contextual relationships in textual data and effectively identify malware-related patterns [9]. Moreover, by decentralizing model training, federated learning enhances malware detection while preserving privacy and adapting to evolving cyber threats [10]. Furthermore, explainable AI (XAI) enhances the transparency of malware detection by making machine learning models more interpretable. Techniques such as SHAP and LIME provide insights into model decisions, helping analysts understand why a file or behavior is classified as malicious or benign [11].

Although previous studies have explored malware detection on individual platforms, there is a lack of comprehensive reviews that thoroughly analyze machine learning techniques across multiple platforms. Multi-platform malware detection is critical for several reasons. First, cyberattacks today often exploit the weakest link across interconnected systems. For example, a single vulnerability in an IoT device can be leveraged to infiltrate broader networks, including enterprise cloud systems. Second, malware has evolved to operate across multiple platforms, with many modern variants designed to adapt to different environments. For instance, Mirai, a botnet that initially targeted IoT devices, was later modified to attack cloud-based systems and enterprise networks. Hence, developing unified defense strategies is essential, as organizations adopt hybrid environments that combine on-premises and cloud-based systems.

This survey aims to fill this gap by providing a holistic review of the recent literature on malware detection using ML methods across diverse platforms. The motivation for this study was also based on my previous work [12], in which we explored and analyzed various modern malware attack trends and defense mechanisms. This study focuses on malware detection across diverse platforms (PCs, mobile devices, IoT systems, and cloud environments), offering a novel perspective and enabling deeper analysis and new insights into the evolving malware landscape. It also outlines the specific challenges encountered on each platform and provides insights for adapting techniques for cross-platform usage. This survey serves as a valuable resource for cybersecurity researchers and practitioners while laying the groundwork for future research on adaptable cross-platform malware detection using machine learning. The key contributions of this study are as follows:

- We conducted a thorough review of the latest literature on malware detection published since 2017, revealing that this is the first comprehensive survey to explore machine learning-based malware detection across PCs, mobile devices, IoT systems, and cloud environments.
- This study investigated platform-specific features (e.g., static, dynamic, memory, and hybrid) for training ML models and analyzed the malware landscape across platforms. It comprehensively reviews ML- and DL-based malware detection techniques and highlights the key research trends for each platform.
- This study identified both platform-specific challenges and cross-platform issues that affect the development of effective ML-based malware detection techniques.
- Finally, this study identifies the limitations of the existing literature and suggests future research directions.

The remainder of this paper is organized as follows: Section 2 presents a comparison of the results of previous related studies. Section 3 provides an overview of malware, including definitions, leading threats, analysis, and features used to build ML detection models. Section 4 describes the malware landscape across diverse platforms. Section 5 presents an overview of machine learning algorithms for malware detection. Section 6 reviews malware detection using ML techniques for PCs, mobile devices, the IoT, and cloud platforms. Section 7 presents the challenges associated with the platform and cross-platforms. Section 8 presents the limitations of the existing literature and future research directions. Section 9 concludes this paper.

## 2. Comparison with Previous Related Surveys

This section examines survey papers on malware detection via machine learning from 2017 onwards, highlighting the gaps that we intend to address. This will help researchers to establish a baseline for developing countermeasures. Table 1 compares our survey with existing surveys.

Existing surveys on malware detection using machine learning and deep learning typically focus on specific platforms such as Windows [13,14,15,16] or Android [17,18,19]. A small number of studies [7,20] have examined both Windows and Android. Some surveys [21,22,23] have addressed malware classification in IoT platforms using ML and DL techniques. However, many current studies lack a comprehensive understanding of the IoT malware. Few studies have focused on cloud malware. Belal and Sundaram [24] provided a taxonomy of ML- and DL-based cloud security, addressing issues, challenges, and trends, whereas Aslan et al. [25] discussed behavior-based malware detection in the cloud. Table 1 also shows that several surveys have focused exclusively on DL technologies for malware detection, such as those in [7,15,26,27], without focusing on traditional ML or ensemble learning techniques. However, traditional ML and ensemble learning offer distinct advantages including lower computational requirements, faster training times, and better performance on smaller datasets.

**Table 1:** Summary of existing review papers for comparison with our study (myth: √ indicates complete information provided, ≈ indicates partial information provided, and × indicates no information provided).

Papers	Year	Main Contribution	Insights into Malware				ML-Based Malware Detection in Diverse Platform					Challenges Identified
			Latest Prominent Malware Variants	Platform-Based Malware Taxonomy	Analysis Methods (Static, Dynamic, Memory, and Hybrid)	Feature Details	PCs		Mobile	IoT	Cloud	
							Windows	Linux				
[13]	2021	Survey on malware detection techniques using machine learning algorithms.	×	×	√	×	√	×	×	×	×	×
[14]	2019	Survey on sophisticated attack and evasion techniques used by the contemporary malwares.	×	×	≈	×	√	×	×	×	×	×
[15]	2022	This survey is on the use of deep learning-based malware detection.	×	×	≈	×	√	×	×	×	×	×
[17]	2021	Reviewed machine learning methods for Android malware detection.	×	×	√	×	×	×	√	×	×	×
[16]	2020	Study on traditional and state-of-the-art ML techniques for malware detection.	×	×	≈	√	√	×	×	×	×	×
[18]	2023	DL approaches for malware defenses in the Android environment.	×	×	√	√	×	×	√	×	×	×
[19]	2020	Android malware detection using deep learning.	×	×	≈	√	×	×	√	×	×	×
[21]	2023	Survey on IoT malware taxonomy and detection mechanisms.	×	≈	×	×	×	×	×	√	×	√
[22]	2023	Discussed IoT dataset use to evaluate machine learning techniques.	×	×	≈	×	×	×	×	√	×	×
[23]	2023	Review on emerging machine learning algorithms for detecting malware in IoT.	×	×	×	×	×	×	×	√	×	×
[7]	2024	Modern deep learning technologies for identifying malware on Windows, Linux, and Android platforms.	×	×	√	≈	√	√	√	×	×	×
[20]	2021	Computer-based and mobile-based malware detection and their countermeasures are presented.	×	×	≈	×	√	×	√	×	×	√
[24]	2022	ML- and DL-based defenses against attacks and security issues in cloud computing is provided.	×	×	×	×	×	×	×	×	√	√
[25]	2021	Behavior-based malware detection system in the cloud environment.	×	×	≈	×	×	×	×	×	×	×
**Our survey**	**2024**	**Survey on malware detection in PCs, mobile devices, IoT, and cloud platforms using ML techniques.**	**√**	**√**	**√**	**√**	**√**	**√**	**√**	**√**	**√**	**√**

These benefits highlight the importance of exploring these techniques along with DL for comprehensive malware detection strategies. Moreover, existing surveys fail to comprehensively address malware detection across platforms such as Linux, macOS, iOS, IoT, and the cloud, which are also frequently targeted by malware. The lack of platform diversity in current surveys highlights the need for an inclusive review that covers various environments to thoroughly understand malware detection methods. To fill these gaps, this study provides a comprehensive survey of recent ML and DL approaches for malware detection across Windows, Linux, macOS, Android, IoT, and cloud platforms, which are frequently targeted by malware.

## 3. Malware Fundamentals

This section explores the fundamental aspects of malware, including its definition, type, and disruptive impact on systems and data. It highlights recent significant malware threats, discusses standard analysis techniques, and examines the critical features that enable machine learning to detect and combat these threats.

### 3.1. What Is Malware?

Malware refers to malicious software designed to compromise systems or gain unauthorized access. Despite advancements in cybersecurity, disruption of systems by stealing data, damaging files, or rendering services unavailable remains a significant threat. Common categories include viruses, worms, trojans, ransomware, spyware, adware, botnets, and rootkits, each with distinct behaviors and objectives. For example, viruses modify or delete files, worms self-replicate across networks, rootkits allow remote control, and trojans masquerade as legitimate applications for covert activities. Adware displays unwanted ads, spyware tracks user activities, botnets exploit resources, and backdoors bypass security for unauthorized access [28]. This classification highlights the diverse operational goals of malware and emphasizes its evolving threat landscape.

### 3.2. Leading Malware Threats in the Current Cyber Landscape

The cyber threat landscape is dominated by sophisticated malware targeting multiple platforms. Malware employs evasive, polymorphic, and adaptive tactics to avoid traditional security measures and pose detection challenges. Cybercriminals also leverage AI-powered malware, which complicates defense. This section provides an overview of prevalent malware threats, emphasizing the need for cybersecurity professionals to remain informed and proactive against these evolving risks.

**Ransomware:** Ransomware remains one of the most widespread and damaging forms of malware. The COVID-19 pandemic has increased ransomware activities, which escalated further in 2023. Attacks have shifted from targeting large enterprises to small businesses through RaaS kits, with LockBit leading this trend. Despite February 2024 server seizures, LockBit 3.0, re-emerged shortly after [29]. Ransomware targets diverse devices, including desktops, mobile devices, IoT, and cloud environments, using phishing and exploits to deliver payloads [12]. Recent ransomware attacks by Conti, REvil, Darkside, and LockBit 3.0 have significantly impacted global infrastructure, healthcare, and businesses. Conti’s attack on Costa Rica’s government led to a national state of emergency [30], while REvil’s Kaseya breach demanded a USD 70 million ransom [31]. Darkside’s Colonial Pipeline incident cost USD 5 million [32]. LockBit 3.0’s Accenture breach demanded a USD 50 million ransom [33].

**Advanced Persistent Threats (APTs):** APTs are sophisticated, targeted attacks designed for espionage or sabotage, projected to drive a USD 12.5 billion market by 2025 [34]. It employs advanced tactics, such as obfuscation, anti-analysis techniques, and AI to evade detection and exploit zero-day vulnerabilities [35]. Their multistage process includes reconnaissance, initial access (e.g., spear phishing), privilege escalation, lateral movement, and data exfiltration while maintaining stealth. Notable examples include Stuxnet, which disrupts Natanz’s centrifuges through zero-day vulnerabilities and fake software signatures [36]. The SolarWinds attack is another example of an APT that deploys malware via a supply chain compromise in an Orion system [35]. These attacks highlight the evolving complexity and persistence of the APTs.

**Figure 2:**
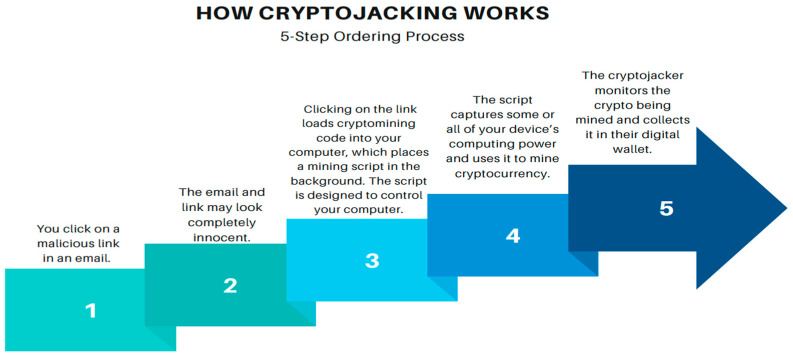
Step-by-step process of cryptojacking [38].

**Cryptojacking:** Cryptojacking is a stealthy cyberattack in which malware is injected via malicious links into a network of devices and runs covertly to harness the victim’s computing resources for mining cryptocurrencies. In 2023, cryptojacking incidents skyrocketed, exceeding the previous year’s total by early April and reaching USD 1.06 billion by the end of the year—a 659% increase [37]. Unlike ransomware, cryptojacking avoids direct payment demands and uses obfuscation to avoid detection. Figure 2 [38] illustrates this process in a step-by-step manner.

Cryptojacking exploits desktops, servers, mobile devices, and cloud platforms, using various forms of malware or scripts for unauthorized cryptocurrency mining. Browser-based cryptojacking exploits devices by using malicious JavaScript. It requires no software installation but may cause increased CPU usage, slowdowns, or device overheating. Host-based methods involve the direct installation of scripts to misuse CPU/GPU resources, whereas cloud cryptojacking exploits server vulnerabilities and causes financial and performance losses. Significant cryptojacking incidents include breaches at a European water utility, Tesla’s cloud, and the Los Angeles Times website in 2018 [37,38]. The U.S. Department of Defense discovered cryptojacking malware in 2020 [39], and a Russian nuclear facility employee was fined USD 7000 for illegal Bitcoin mining in 2019 [40].

**Spyware:** Spyware, like Pegasus, infiltrates networks to steal sensitive data such as login credentials, screenshots, and chat histories. It exploits BYOD policies to access mobile devices, compromising emails, SMS, app data, and multimedia. Pegasus can bypass multi-factor authentication by extracting one-time passwords [29].

**Wiper malware:** Wipers are malicious programs that destroy user data and target computer networks. Threat actors use wipers to conceal intrusions and to hinder responses. Nation-state attackers deploy them to disrupt supply chains and military operations, while “hacktivists” use them to impede business activities in response to perceived injustices [41]. Recent examples include WhisperGate, targeting Ukraine in January 2022 [42], and HermeticWiper, which affected Ukrainian organizations in February 2022 [41].

**Remote access trojans (RATs):** RATs, a specific trojan type, are popular with cybercriminals for remotely controlling endpoint devices. They trick users to run malicious code by masking them as legitimate applications. Ghost, a remote access trojan, controls infected endpoints. Unlike typical malware, Ghost is deployed manually, suggesting that victims are already compromised by other malware [29]. Understanding these threats is key to advancing countermeasures and detection.

### 3.3. Malware Analysis

In this subsection, we explore the key malware analysis methods essential for malware detection systems, focusing on identifying suspicious file characteristics and purposes.

- Static analysis;
- Dynamic analysis;
- Memory analysis;
- Hybrid analysis.

Static analysis techniques extract static signatures, features, or patterns from binary files without execution. This method is fast, secure, and efficient in identifying known malware samples, and does not require kernel privileges or a virtual machine. However, static analysis has significant limitations: it cannot examine malware strains using obfuscation techniques and is ineffective against malware that uses packers to compress and encrypt payloads [43].

Conversely, dynamic analysis involves executing malware in a controlled environment to observe runtime behavior. This enhances the understanding of malware functionality and enables the identification of previously unknown or zero-day malware. However, this approach is often slower and more time-consuming [43]. Additionally, dynamic analysis also has limitations in tracking highly sophisticated malware, such as fileless (memory-resident) malware.

Consequently, memory analysis offers an alternative method for detecting malicious behaviors of fileless malware by capturing and examining volatile memory images during execution. While encryption and packing can conceal suspicious files, all processes are visible in the memory during runtime. Malware must disclose critical information (e.g., logs, code, and data segments) for operational functionality, making detection possible. Volatile memory analysis detects malware by examining its presence in the system’s RAM and identifying fileless malware that evades detection by not leaving traces on hard drives [44].

The hybrid malware analysis methodology combines multiple analysis approaches, offering greater effectiveness than a single analysis technique.

### 3.4. Features Used in ML-Based Malware Detection

This subsection provides an overview of the features extracted from various platforms, each of which uses distinct file formats and yields different features. In Windows, malware features are extracted from executable (EXE) files, whereas Linux malware is analyzed using Executable and Linkable Format (ELF) files. In macOS, the Mach-O file format is used to analyze and extract the features. Android relies on APKs, and iOS utilizes IPA files. The APK file enables the extraction of static features from classes.dex files and dynamic features from the AndroidManifest.xml file [45]. IoT platforms derive features from firmware binaries, whereas cloud environments use container images and VM disk files, such as Docker and VMDK, for feature extraction. Table 2 classifies platform-specific features into static, dynamic, and memory-based features suited to different file formats and operating environments. Static features derived from binaries or metadata without execution include file headers, opcode sequences, and metadata, which are essential for assessing executables and packages on Windows, Linux, macOS, Android, and iOS. Dynamic features capture behavior during execution, including system calls, API invocations, network activities, and registry or file system changes, aiding in the identification of complex or evasive malware. Memory features, such as memory allocation patterns and mapping, are vital for detecting sophisticated threats, particularly in IoT and cloud environments. This structured feature analysis underpins the implementation of machine learning models attached to each platform’s unique characteristics.

## 4. Malware Landscape Across Platforms

**Figure 3:**
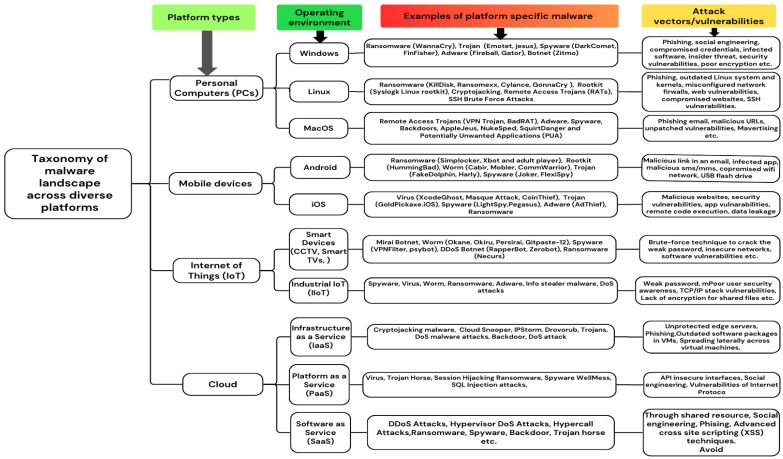
Taxonomy of malware landscape across various platforms.

The proliferation of digital technologies has expanded the malware threat landscape across PCs, mobile devices, the IoT, and cloud systems. Understanding the targeted operating system or device is crucial for comprehending malware behavior, as malicious software often exploits system-specific vulnerabilities. In this study, “platform” and “operating system” will be used synonymously, and we classify the target platforms for malware into four primary categories: PCs, mobile devices, IoT, and cloud systems. Each platform has unique vulnerabilities, attack vectors, and security issues, which require distinct detection and mitigation strategies. This section provides an overview of the malware landscape across these platforms, as shown in Figure 3.

### 4.1. PCs

The PC platform is a primary target for malware, facing various types that exploit specific vulnerabilities in Windows, macOS, and Linux environments. This study examines the malware landscape for each operating system, emphasizing common threats, typical attack vectors, and mitigation mechanisms.

#### 4.1.1. Windows

The Windows platform remains a primary malware target owing to its extensive use in personal and enterprise settings. Malware types include viruses, worms, trojans, ransomware, spyware, adware, and rootkits, which threaten the system’s integrity and data security. Cybercriminals exploit phishing emails, malicious websites, software vulnerabilities, and removable media for infections. Advanced techniques such as polymorphism, obfuscation, and encryption avoid traditional detection, thus necessitating adaptive detection mechanisms [14]. The platform’s broad software ecosystem provides numerous attack entry points. Although Microsoft employs security measures, such as Windows Defender and regular updates, their effectiveness depends on users practicing safe computing and maintaining updated systems. The impact of malware on Windows can lead to system performance issues, data theft, crashes, and financial losses, highlighting the significant consequences of these attacks.

#### 4.1.2. Linux

Linux has become the leading operating system in multi-cloud environments, powering 78% of the world’s top websites. This widespread use has increased the scale and complexity of Linux-based systems [48]. The Linux OS supports various distributions for diverse hardware needs, is integral to many Internet-based desktop devices, and is a target for cybercriminals. The rise in Linux-based malware attacks is due to IoT devices running Linux-based firmware such as smart home assistants, security cameras, and industrial control systems. These devices often lack robust security, which makes them vulnerable to attacks. As more companies adopt Linux-based servers and networks, hackers are increasingly targeting these systems for greater rewards. Trend Micro’s research shows that 90% of public cloud workloads run on Linux, motivating hackers to develop Linux malware [49]. Recently, Linux-based systems have been facing an increase in malware attacks. According to the VMware threat report [48], these devices face cryptojacking malware, remote access trojans (RATs), SSH brute-force attacks, web shell malware, and ransomware. The Trend Micro Linux Threat Landscape Report indicated a 62% increase in ransomware attacks on Linux from 2022 to 2023. It identified that the KillDisk Ransomware targeted financial institutions, exploited phishing attacks, and outdated Linux systems and kernels. This report also states that Webshell exploits are the most common Linux malware at 49.6%, followed by trojans at 29.4%, whereas backdoors and cryptominers are less prevalent [49]. Cybercriminals primarily exploit web vulnerabilities such as SQL injection, XSS, and SSRF to compromise web resources. They also targeted cloned websites, misconfigured firewalls, and SSH vulnerabilities to execute malware attacks on Linux systems.

#### 4.1.3. macOS

The evolving macOS threat landscape necessitates greater vigilance from users and developers. Despite the robust security reputation of macOS, it remains vulnerable to cyber threats in 2022; malware detection on macOS rose by 165%, accounting for 6.2% of the total increase from the previous year [50]. MacOS employs security features like XProtect and Gatekeeper, but they have limitations. XProtect’s signature-based detection is ineffective against unknown or modified malware and lacks dynamic scanning capabilities. The gatekeeper blocks unsigned or malicious Internet applications, verifies developer IDs, and checks for alterations after signing. However, attackers can bypass this using stolen developer IDs or exploit legitimate apps to run malicious codes. While sandboxing applications limit access to vital system resources, attackers have devised techniques to escape and obtain illicit access [50]. The most prevalent macOS malware include adware, potentially unwanted programs (PUPs), backdoor spyware, remote access trojans, stealers, ransomware, and other emerging types [50,51]. New threats have emerged, such as AppleJeus, which shifted from Windows to macOS in 2018, and NukeSped, which functions as ransomware, spyware, and stealer, detected in 2019. SquirtDanger, a macOS-targeting malware with advanced evasion techniques, was discovered in 2022 [50]. Common attack vectors include malvertising, phishing emails, malicious URLs, and unpatched vulnerabilities with persistent macOS vulnerabilities.

### 4.2. Mobile Devices

The increasing prevalence of mobile devices has made them prime targets for malware, particularly for smartphones. Malware developers primarily target Android and iOS operating systems, which dominate the global mobile OS market.

#### 4.2.1. Android

The widespread adoption of Android platforms on smartphones, tablets, and IoT devices has increased their vulnerability to cyberattacks. The flexibility, cost-effectiveness, and computing power of Android devices have increased in popularity. They offer user-friendly third-party applications that are globally accessible via Internet connections. This popularity has made Android susceptible to cyberattacks. A recent report revealed that over 438,000 mobile malware installation packages were detected in Q3 2023, marking a 19% increase from Q2 [52]. Another report showed that in Q2 2024, Android led the global mobile market with a 71.65% share, whereas iOS accounted for 27.62% [53]. Android platforms face malware threats including credential theft, privacy breaches, bank fraud, ransomware, adware, and SMS fraud. The development of automatic Android malware detection methods is vital for protecting system security and user privacy.

Android is an open-source Linux-based mobile OS that allows anyone to access its own code. Its architectural framework comprises distinct layers: a kernel, hardware abstraction layer, Android runtime, libraries, application framework, and applications. These components optimize the system efficiency and application performance. Android offers security mechanisms such as sandboxing, permissions, and encryption to protect data and ensure app integrity [54]. Android apps operate in isolated sandboxes with user-approved permissions for resources like cameras and Wi-Fi. Users should exercise caution when granting permission because malicious apps can access sensitive resources once allowed.

Various forms of malware, such as SMS trojans, ransomware, adware, backdoors, rootkits, spyware, botnets, and installer malware, significantly threaten mobile device security [55,56]. Malware spreads on mobile devices through malicious links in emails or SMS, infected apps from Google Play Stores, third-party sources, or malicious Wi-Fi networks. Significant vulnerabilities in the Android OS include information gain, code execution, denial of service (DoS), overflow, SQL injection, and privilege escalation [56].

#### 4.2.2. iOS

iOS, introduced in 2007, is a Unix-based operating system powering Apple devices, such as iPhones and iPads, ranking second in global mobile OS usage. The iOS architecture comprises four layers: the Core OS handles hardware interactions, Core Services provide data protection and storage, media support multimedia processing, and Cocoa Touch enables app development and user interface management [56].

iOS offers robust security compared with Android through a closed system design incorporating device-level protection (e.g., PINs, remote wipe), system-level features (e.g., Secure Enclave, secure boot), and mandatory data encryption. Apple’s control over hardware and software makes jailbreaking and unauthorized access challenging. The iOS enhances security through sandboxing, encryption, and automatic data erasure. Applications are isolated to prevent unauthorized access, whereas encryption protects files using hardware and software keys [57]. iOS automatically grants most permissions, thereby reducing user involvement. The auto-erase feature wipes data after multiple incorrect passcode attempts, offering higher security than Android [58]. A McAfee report shows iOS malware surged by 70% by 2020 [56]. Common malware on iOS includes ransomware, spyware, viruses, trojans, and adware [56,59]. Notable attacks include Pegasus, exploiting zero-day vulnerabilities for surveillance [60], and LightSpy Spyware infiltrated via compromised news sites [61]. Vulnerabilities, such as memory overflow, remote code execution, and data leakage, present significant risks to iOS users, highlighting the need for enhanced device security.

### 4.3. IoT Platform

The Internet of Things (IoT), introduced by Ashton in 1999, refers to a network of interconnected devices that collect and exchange data via the Internet or other networks. These include devices, sensors, networks, computing resources, and software tools. IoT devices fall into two categories: Consumer IoT, such as personal and wearable smart devices, and Industrial IoT (IIoT), including industrial machinery and energy management devices.

The number of IoT devices is increasing significantly each year. According to Statista, global IoT devices will nearly double from 15.9 billion in 2023 to over 32.1 billion by 2030. By 2033, China will have the highest number of IoT devices, with approximately eight billion consumer devices [62]. However, the rapid rise of IoT coupled with insufficient security measures has made these devices prime targets for malware. Reports from Zscaler ThreatLabz show a 400% increase in IoT malware attacks [63]. High-profile incidents, such as the Mirai botnet in 2016, exploit weak passwords and unpatched vulnerabilities, thereby enabling DDoS attacks and data exfiltration [63]. IoT malware also takes advantage of other vulnerabilities, including the absence of software and security updates, insecure networks, poor user security awareness, TCP/IP stack vulnerabilities, and a lack of encryption. Modern IoT malware, including Okane, VPNFilter, and Necurs, increasingly employs brute-force methods, spyware tactics, and anti-virtualization techniques to access devices [21].

### 4.4. Cloud Environments

Cloud computing enables remote access to computing resources such as storage, applications, networks, and servers via an Internet connection. Conversely, cloud malware is a cyberattack that targets cloud platforms with malicious code or services.

Cloud computing offers three types of services: Platform as a Service (PaaS), Software as a Service (SaaS), and Infrastructure as a Service (IaaS). PaaS provides an environment for programmers to develop, deploy, and test applications, as exemplified by the Azure and Google App Engine. SaaS supports all applications within the cloud environment such as email and office software. IaaS offers hardware resources, computing capabilities, storage, servers, networking devices, and virtual machines [24,64]. Common examples of cloud malware include DDoS attacks, hypervisor DoS attacks, hypercall attacks (exploits the hypervisor to gain cloud control), hyperjacking (when an attacker takes control of a virtual machine for malicious purposes), exploiting live migrations (moves a VM or application without client disconnection from one physical location to another), ransomware, spyware, backdoor, trojan horse etc. [24,65].

## 5. Machine Learning Algorithms for Malware Detection

In this section, we present a summary of various machine learning algorithms used for malware detection on diverse platforms, including traditional, ensemble, and deep learning approaches, as outlined in Table 3.

Traditional algorithms such as SVM, KNN, and DT are simple yet effective in classifying malicious and benign samples. Ensemble methods, such as RF and gradient boosting, enhance the accuracy and robustness by combining multiple models. Deep learning algorithms, including CNN and transformers, excel in processing complex, high-dimensional, and sequential malware data. Techniques such as GAN and Transfer Learning (TL) address challenges such as limited datasets and feature extraction. Moreover, recent advancements in federated learning (FL) and large language models (LLMs) have shown promise for malware detection.

**Figure 4:**
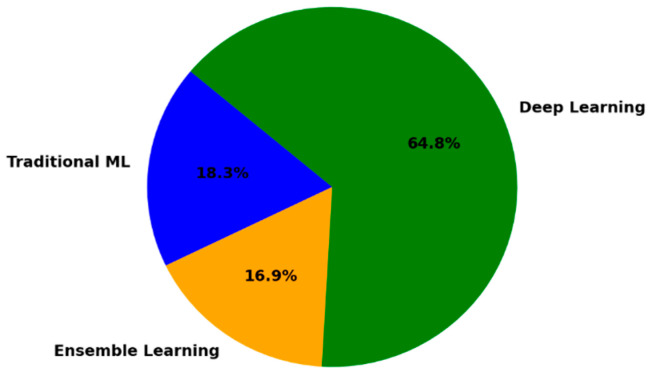
Proportion of algorithm categories in recent malware detection.

Table 3 underscores the diversity of machine learning methodologies in malware detection. The analysis outcomes of the table are reflected in the pie chart shown in Figure 4, which highlights the key trends in machine learning techniques for malware detection.

**Overall research trends on machine learning algorithms for malware detection across different platforms**

Table 3 and the pie chart reveal that deep learning is the leading approach in malware detection research across platforms, with CNNs and LSTMs excelling in image-based and sequential data analysis. Traditional ML techniques such as SVM and KNN remain adequate for high-dimensional feature-based tasks. Simultaneously, ensemble learning methods, such as random forest and gradient boosting, show substantial accuracy and generalization through model aggregation. These trends highlight the increasing preference for deep learning, while acknowledging the complementary roles of traditional and ensemble models.

## 6. Application of Machine Learning on Malware Detection

This section reviews recent studies that have utilized the various ML algorithms discussed in Section 5 to develop malware detection models for Windows, Linux, Android, IoT, and cloud platforms.

### 6.1. PC (Personal Computers) Malware Detection

This section covers malware detection on personal computers, including Windows, Linux, and macOS. Windows, the most widely used OS, is the primary target for malware. Despite Linux’s robust permission-based architecture, it is facing growing threats in the server and enterprise settings. With its increasing popularity, macOS has increased the risk of malware. Detection methods employ static, dynamic, and hybrid analyses, which are frequently enhanced using machine learning, to counter evolving threats.

#### 6.1.1. Malware Detection on Windows Platform

In this subsection, we provide an extensive review of machine learning-based malware detection techniques for the Windows platform, which are summarized in Table 4.

**Static feature-based malware detection techniques**

Jeon and Moon [75] proposed a DL-based malware detection method using static opcode sequences with dynamic RNN and CNN. A convolutional autoencoder compresses long opcode sequences, and a recurrent neural network classifies malware using the features generated by the autoencoder. This method achieved 96% accuracy and a 95% true positive rate. However, it requires substantial computational resources owing to the inter-procedural control-flow analysis, making it less suitable for resource-limited systems. Snow et al. [76] developed a multimodal deep-learning-based malware detection method using the Microsoft BIG 2015 dataset. Although the model achieved a high accuracy rate of 98.35%, it proved ineffective against zero-day malware that evaded detection using new static signatures. A previous study [104] employed a CNN-based pre-trained DenseNet model for malware detection by converting benign and malicious binaries into 2D images. Experiments on Malimg, BIG 2015, MaleVis, and Malicia datasets showed 98.23% accuracy on Malimg but revealed high false negatives and used highly imbalanced datasets. Darem et al. [77] implemented a deep CNN-based model to detect malware using opcode-level features from malware and benign binary files that were converted into images for training. The model achieved a detection rate of 99.12% using the Microsoft BIG 2015 dataset. However, outdated datasets can affect the detection of new and unseen malware. Kumar and Janet [105] proposed an image-based deep transfer learning model for detecting Windows malware using a pre-trained deep CNN. The model efficiently extracts high-level features from grayscale images of Windows executables, conserving resources and time; however, it struggles to identify malware packed using advanced techniques. A study in [109] introduced an attention-based deep neural network (ATT-DNN) for malware detection, extracting static features from executable files. Despite achieving a high accuracy of 98.09%, its use is limited to malware detection based on static signatures. Studies [78,79,110] have also focused on malware detection via static image analysis.

**Dynamic feature-based malware detection techniques**

Li et al. [94] developed a DL model for malware detection in executables using API call sequences within a Cuckoo sandbox, achieving an F1-score of 0.9724 and 97.31% accuracy on new sequences. The limitations of this study include its focus on Windows 7 executables and the potentially reduced effectiveness against zero-day malware over time. In [111], contextual analysis of API call sequences was utilized to enhance the dynamic detection and prediction of Windows malware, thereby improving both the accuracy and adaptability to evolving threats. By employing the Markov chain method, they achieved an average accuracy of 99.7%. Catak et al. [112] proposed an LSTM-based malware detection method that achieved 95% accuracy and 0.83 F1 score using a behavioral dataset of API calls. They also released a new, publicly available API call dataset for malware detection. Aditya et al. [95] used LSTM and GRU deep learning models to classify malware based on API call sequences, and achieved an accuracy of 96.8% with LSTM. However, their dataset was highly imbalanced, with only 1035 benign samples of 8142. In [66], a hybrid framework combining multiple complementary filters with a wrapper feature selection method was proposed to identify the critical runtime behavioral traits of malware. The ML algorithms, including MRed, SVM, and Fisher, achieved a detection accuracy of 99.499%. Ring et al. [96] used an LSTM-based model to detect malware based on audit log features. However, it suffers from high false positive rates and lacks the evaluation of larger datasets to assess the model’s scalability. Jindal et al. [80] proposed Neurlux, a stacked ensemble of CNN-LSTM with an attention mechanism to detect malware in Windows systems using dynamic features effectively but is susceptible to adversarial attacks.

**Hybrid feature-based malware detection techniques**

Hybrid feature-based learning approaches have shown promise in cybersecurity, outperforming single-type feature methods. By combining diverse feature types, such as static, dynamic, and memory-based, these techniques enable learning from multiple semantic perspectives, leading to an enhanced model accuracy for malware detection and classification.

The authors of article [81] created a CNN-based hybrid malware classification model for Windows, integrating static features from the executable section, static API call imports, dynamic API calls, and executable file images, achieving a detection accuracy of 97%. However, this method does not validate the effectiveness of the combined feature sets against adversarial attacks. AI-HydRa [73] represents an advanced hybrid malware classification method that combines RF and deep learning, achieving a mean detection rate of 0.851 with a standard deviation of 0.00588 for the three tests. Huang et al. [106] introduced a hybrid method using static images and dynamic API call sequence visualizations to classify malicious behaviors. Utilizing a CNN (VGG16) for feature extraction, the technique attained 94.70% accuracy but had difficulty accurately identifying specific malware types, including password-stealing (PSW) trojans and some outdated variants. RansHunt [113] integrated static and dynamic features for improved ransomware detection using an SVM, achieving an accuracy of 97.10%, outperforming traditional antivirus solutions. Darabian et al. [82] used static and dynamic features from 1500 cryptojacking malware samples. They used opcodes and system calls to construct CNN, LSTM, and attention-based LSTM classification models, achieving 95% accuracy in static analysis and 99% accuracy in dynamic analysis.

Karbab et al. [114] introduced SwiftR, an approach that detects ransomware attacks by integrating various static and dynamic features from benign and malicious executable file reports. The proposed method achieved an F1 score of 98%.

**Memory feature-based malware detection techniques**

Malware detection using static or dynamic analysis is insufficient for advanced memory-resident malware and obfuscated malware. Thus, contemporary research emphasizes memory analysis methods that are effective for detecting sophisticated malware variants [44]. The study [83] developed a CNN model was developed that used memory images of both suspicious and benign processes to detect malware attacks with a detection rate of 98%, leveraging features extracted from gray-level co-occurrence matrices and local binary patterns. Another study [84] proposed a DL-based approach that integrated GAN and CNN models, achieving 99.60% detection accuracy on unseen samples when tested on the DumpWare10 dataset to identify advanced malware by visualizing the running processes. In addition, Naeem et al. [85] developed a high-performance stacked CNN and MLP model using memory images and achieved an accuracy of 99.8%. However, it has limitations in terms of the training time complexity and susceptibility to adversarial attacks. In [70], the authors used the latest dataset, the CIC-MalMem-2022 dataset, to develop a CNN-based detection model that detects obfuscated malware in memory.

**Summary of key trends and insights on malware detection on the Windows platform**

**Figure 5:**
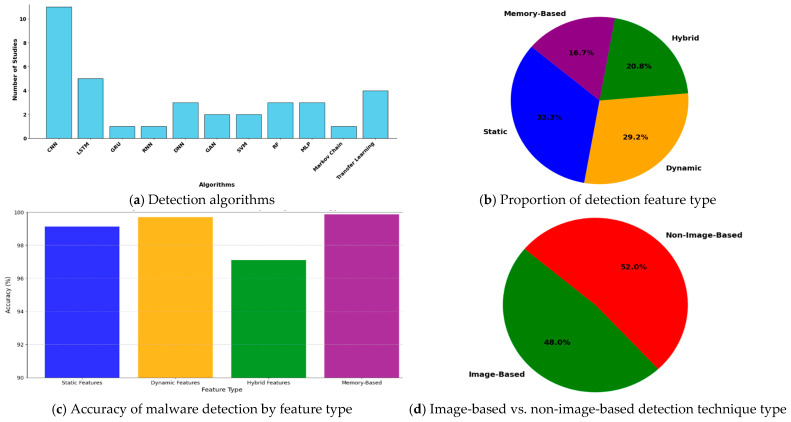
Distribution of detection feature types, algorithms, accuracy by feature type, and image and non-image features.

A summary of the malware detection methods for Windows is presented in Table 4. The table outlines studies with respect to their data collection source, feature type, features, ML algorithms used, detection accuracies, and limitations. Figure 5 illustrates the distribution of the techniques, features, and evaluation datasets used in these studies.

**Dataset evaluation:**Table 4 shows that many recent studies have relied on outdated datasets, such as Malimg (2011) and Microsoft BIG 2015. VirusTotal and VirusShare remain the most popular data sources for Windows malware detection systems, followed by dumpware10 and Hybrid Analysis.com. In contrast, newer datasets such as CIC-MalMem-2022 provide updated benchmarks. However, the prevalence of outdated datasets and the lack of diversity in recent studies have limited their effectiveness against zero-day malware and emerging threats.

**Detection algorithms used in the studies:** The bar chart in Figure 5a illustrates the distribution of detection algorithms employed in various detection techniques, highlighting their relative popularity among the studies. Convolutional neural networks (CNNs) are the most-utilized algorithms, likely because of their efficiency in handling images and spatial data. LSTM networks also stand out and are likely to be favored because of their strength in temporal or sequential data processing. Algorithms such as GANs and transfer learning are used less frequently and hint at emerging or specialized applications. However, GRU, RNN, and Markov chains appear less favored, possibly because of their limited generalizability or lower performance in detection tasks. The chart collectively underscores the significance of choosing algorithms that align with the nature of the problem and the data characteristics.

**Detection feature type:** Figure 5b highlights the distribution of feature types in the malware detection techniques. Static features lead by 33.3%, favoring their simplicity and effectiveness against known malware. Dynamic features follow closely at 29.2%, offering strong runtime analysis capabilities but requiring controlled execution environments. Hybrid features, at 20.8%, integrate static and dynamic methods for comprehensive detection but involve higher computational demands. Memory-based features, representing 16.7%, are powerful for analyzing runtime data, such as API calls, but are less commonly used because of their resource-intensive nature.

**Accuracy of malware detection techniques by feature type**: The bar chart in Figure 5c compares the accuracy of malware detection techniques based on four feature types: static, dynamic, hybrid, and memory. Memory-based features achieved the highest accuracy (~99.89%), demonstrating their effectiveness in capturing runtime behaviors, although they may require higher computational resources. Dynamic features also perform well (~99.49%), leveraging runtime analysis, whereas static features (~99.12%) offer robust results through code and signature analysis. Hybrid features (~99%) combine static and dynamic methods but do not significantly outperform individual approaches. Overall, memory-based and dynamic features demonstrate the highest potential for accurate malware detection.

**Image-based vs. non-image-based detection techniques:** The pie chart in Figure 5d shows a close competition between non-image-based (52%) and image-based (48%) detection methods. While non-image-based methods lead slightly because of their flexibility with diverse data types, image-based approaches are emerging as powerful tools in malware detection. By converting malware binaries into images, image-based methods use CNNs to analyze spatial patterns and effectively identify complex obfuscated malware. The availability of labeled malware datasets, efficient pre-trained models, and generalization capabilities further drive their adoption, reflecting the growing significance and scalability of image-based methods in modern malware detection.

#### 6.1.2. Malware Detection on Linux OS

Researchers have also utilized various ML algorithms to detect malware attacks on Linux systems. The ML-based Linux detection techniques are listed in Table 5. Xu et al. [107] developed a graph-based Linux malware detection system called HawkEye that achieved 96.82% accuracy. Hwang et al. [115] also demonstrated the effectiveness of deep learning for Linux threat detection, using a large dataset of 10,000 malicious and 10,000 benign files to train and test a DNN model. Bai et al. [74] proposed a Linux malware detection method that analyzes system calls in ELF executable symbol tables using 756 benign and 763 malware ELF samples. They achieved up to 98% accuracy with various classifiers, including J48, random forest, AdaBoostM1, and IBk. Landman and Nissim’s Deep-Hook [86] used CNNs to analyze VM-captured memory dumps and identify Linux malware with up to 99.9% accuracy. Similarly, another study [46] classified malware using behavioral features from volatile memories.

**Summary of key trends on malware detection on Linux platforms**

Most current studies have focused on malware detection in Windows and Android platforms, with few addressing advanced ML-based malware detection on Linux. According to the current literature, Linux-based malware detection has advanced through the integration of diverse machine learning algorithms, feature types, and datasets, achieving high accuracy. Memory-based detection, in particular, has gained popularity owing to its effectiveness in identifying sophisticated threats. Owing to the widespread adoption of Linux OS in online supercomputers and devices globally, cybercriminals have increasingly targeted Linux-based devices. Thus, the success of deep learning in Windows and Android indicates its potential for Linux malware detection. Additionally, exploring hybrid models and cross-platform techniques could enhance the detection capabilities and adapt to the evolving landscape of Linux malware.

#### 6.1.3. Malware Detection on macOS

Despite the rising threats of OS X malware, research on its detection remains scarce, with only a few studies focusing on malware detection on the macOS platform. For example, [116] proposed OS X malware and rootkit detection by analyzing static file structures and tracing memory activities. Pajouh et al. [117] developed an SVM model with novel library call weighting for OS X malware detection, attaining 91% accuracy on a balanced dataset. SMOTE-enhanced datasets increased the accuracy to 96%, with slight false alarm increases, indicating that larger synthetic datasets enhance accuracy, but may impact false positive rates.

**Summary of key trends on malware detection on the macOS platform**

The application of machine learning to OS X malware detection is underexplored, likely owing to the scarcity of suitable datasets and the difficulty in collecting malware samples. Future research should focus on overcoming these challenges to enhance machine learning techniques for detecting OS X malware.

### 6.2. Malware Detection on Mobile Platforms

The increase in mobile device usage, mainly Android, has led to increased malware threats. This section reviews machine learning techniques for detecting malicious applications on both Android and iOS platforms.

#### 6.2.1. Android Malware Detection

This subsection examines ML-based Android malware detection techniques categorized by APK file features, with the dataset details summarized in Table 6.

**Static feature-based malware detection techniques**

A study [118] proposed GDroid, a graph convolutional neural network model for detecting Android malware through API call patterns using static analysis. Although effective in detecting malicious apps, its accuracy decreases in real-world Android devices. Pektaş and Acarman [87] proposed a CNN- and LSTM-based model using static features, such as opcodes, API calls, and call graphs, for Android malware detection. Despite the 91.42% accuracy and 91.91% F-measure for unknown samples, the model’s reliance on static features may limit its effectiveness against obfuscated malware. Similarly, [71] proposed H-LIME, an XAI method that enhances LIME with an opcode-sequence hierarchy for better Android malware detection explanations. Evaluated on the MalDroid-2020 dataset, H-LIME outperformed LIME in explanation quality and efficiency but struggled with shorter programs in real-world malware. Lakshmanarao and Shashi [97] developed an LSTM-based malware detection model using opcode sequences from Android apps and achieved 96% accuracy on a dataset of 1500 apps. Potha et al. [72] created an ensemble model combining LR, MLP, and SGD, showing that larger, homogeneous ensembles with feature selection outperformed smaller ensembles, achieving strong AUC and accuracy on Android malware datasets. Aamir et al. [88] introduced the AMDDL model, achieving 99.92% accuracy in malware detection using CNNs. This study highlights the challenges related to limited malware diversity, deep learning interpretability, and scalability.

**Dynamic feature-based malware detection techniques**

Ma et al. [98] proposed Droidect, a Bi-LSTM-based model for classifying malicious Android apps, achieving 97.22% accuracy on a dataset of 11,982 benign and 9616 malicious files. Despite its success, this model suffers from long detection times. Wang et al. [119] presented a malware detection technique employing network traffic analysis and the C4.5 algorithm, achieving a 97.89% detection rate on the Drebin dataset, outperforming current methods. The study in [99] introduced MemDroid, an LSTM-based detection method trained on Androzoo malware samples. Apps were run in a sandbox to capture system call sequences, which were used to train the LSTM classifier, achieving 99.23% malware detection accuracy. The study in [100] used LSTM to develop classifiers for detecting Android malware via dynamic API and system calls, achieving F1-scores of 0.967 and 0.968, respectively, across different datasets.

**Hybrid feature-based malware detection techniques**

Alzaylaee et al. [67] introduced DL-Droid, a deep learning framework for Android malware detection using static and dynamic analysis. They achieved 97.8% detection with dynamic features and 99.6% detection with combined features, taking 190 s/app. Saracino et al. [55] introduced MADAM, an Android malware detection system analyzing kernel-, application-, user-, and package-level features. MADAM detected over 96% of malicious apps in a 2800-app test but is susceptible to mimicry attacks and cannot identify unknown malware. Wu et al. [101] presented DeepCatr, a hybrid learning approach for Android malware detection, combining text mining and call graphs with bidirectional LSTM and graph neural networks, achieving 95.94% and 95.83% accuracy on 18,628 samples. Mahdavifar et al. [103] created a semi-supervised deep learning model for Android malware detection, using a stacked autoencoder trained on hybrid features, obtaining 98.28% accuracy and a 1.16% false positive rate.

**Memory feature-based malware detection techniques**

Memory analysis has been utilized to develop deep learning models for detecting obfuscated and memory-resident Android malware. A framework combining weak learners (CNNs) and a meta-learner (MLP) to create a deep-stacked ensemble model along with an explainable AI approach for result interpretation and validation was proposed by Naeem et al. [85] The framework achieved an accuracy of 94.3% using 2375 images in an empirical evaluation.

**Summary of research trends on malware detection on the Android platform**

**Figure 6:**
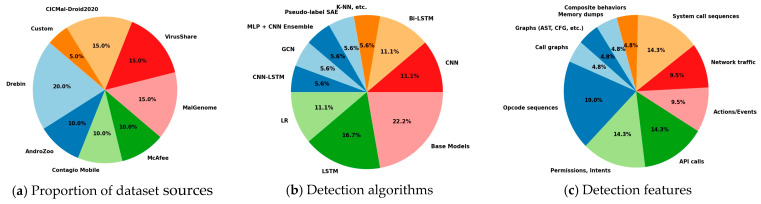
Distribution of detection techniques, detection features, and evaluation datasets used in mobile malware detection solutions.

Table 6 summarizes mobile device malware detection systems, including datasets, features, detection algorithms, and study accuracy. Figure 6 shows the distribution of the dataset sources, techniques, and features used in these studies.

**Proportion of datasets used:** The pie chart in Figure 6a reveals a clear preference for established datasets in Android malware detection studies. Drebin emerges as the most-used dataset (31%), owing to its extensive malware diversity and widespread acceptance as a benchmark in the field. Medium-utilized datasets, including MalGenome, VirusShare, and CICMal-Droid2020 (23% each), are valued for their reliability and growing prominence in the evaluation of detection techniques. The relatively low adoption of custom datasets highlights the focus on standardized datasets, limiting opportunities for novel malware detection approaches tailored to evolving threats.

**Detection algorithms:** The pie chart in Figure 6b demonstrates that the base models (31%), including Logistic Regression and Random Forest, are the most commonly used detection algorithms, valued for their reliability, simplicity, and ease of implementation. Deep learning methods, such as LSTM (23%) and CNN (15%), are gaining popularity owing to their ability to process complex and large-scale malware patterns effectively. Hybrid techniques (8%), ensemble models (8%), and exploratory approaches, such as Pseudo-label SAE and K-NN (8% each), showcase ongoing innovations aimed at improving detection accuracy and robustness. This distribution underscores the balance between traditional dependable methods and modern complexity-driven approaches to malware detection.

**Detection features:** As shown in Figure 6c, the chart indicates that opcode sequences (31%) are the most commonly used features because of their effectiveness in static analysis. Permissions, intents, and API calls (23% each) are essential for identifying behavioral anomalies. System call sequences (23%) and network traffic (15%) are gaining prominence in runtime analyses. Composite behaviors and memory dumps (8% each) remain underexplored, likely due to their complexity and resource demands.

#### 6.2.2. Malware Detection in iOS

In [120], the authors focused on identifying iOS malware using static analysis and machine learning, achieving a high precision of 0.971 and a recall of 1.0. It addresses the underexplored domain of iOS malware detection owing to the platform’s closed-source nature. Zhou et al. [121] examine the risks of legitimate applications being hijacked for malware communication. They presented the ChanDet model to identify potential channel applications and proposed mitigation strategies. Mercaldo and Santone [122] successfully classified 50,000 Android and 230 iOS malware samples using deep learning on grayscale images of executables, tackling obfuscation and false positives.

**Summary of research trends on malware detection on the iOS platform**

The current literature highlights advancements in iOS malware detection, leveraging machine learning and static analysis to address the platform’s closed-source challenges. Researchers have introduced high-precision models and deep learning techniques, such as those using executable images, to mitigate obfuscation. Despite these advancements, challenges such as limited datasets, lack of hybrid analysis, and insufficient attention to real-time cross-platform threats persist. Future work should focus on expanding the datasets, utilizing transfer learning, enhancing anti-obfuscation methods, and developing comprehensive detection frameworks.

### 6.3. Malware Detection on IoT Platform

This section compares the surveys on machine learning-based malware detection in IoT, which are summarized in Table 7.

Ali et al. [68] used machine learning algorithms on the IoT-23 dataset to detect IoT network anomalies. The RF algorithm demonstrated the highest efficacy, achieving 99.5% accuracy. Sudheera et al. [89] introduced Adept, a distributed framework that detects and classifies attack stages in IoT networks through local anomaly detection, pattern mining for correlated alerts, and machine learning-based classification. This method can identify five times more attack patterns with 99% accuracy in classifying attack stages. Vasan et al. [90] proposed a cross-architectural malware detection method suitable for diverse IoT processor architectures, such as MIPS, PowerPC, and SPARC.

Researchers have used sandboxing as a dynamic method to detect malware in IoT environments. However, existing sandboxes are inadequate for resource-limited IoT settings, lack support for diverse CPU architectures, and do not offer library-sharing options [123]. Hai-Viet et al. [91] proposed an IoT botnet detection approach using system call graphs and a one-class CNN classifier, which improved sandboxing to capture system behaviors and utilized graph features for robust detection, overcoming dataset imbalance and architectural constraints, attaining 97% accuracy. Jeon et al. [102] introduced HyMalD, a hybrid IoT malware detection method using Bi-LSTM and SPP-Net to analyze static and dynamic features, extracting opcode and API call sequences for classification. It achieved 92.5% accuracy, surpassing the 92.09% accuracy of the static analysis. Researchers have now converted network traffic or opcode into 2D images for malware detection using visual methods. Shire et al. [92] utilized visual detection techniques in IoT malware detection, transforming network traffic into 2D images for machine learning analysis. He et al. [69] proposed an efficient and scalable lightweight IoT intrusion detection method utilizing feature grouping, which attained over 99.5% accuracy on three public IoT datasets while consuming fewer computational resources than baseline methods. Jiang et al. [93] proposed FGMD, a framework that protects IoT intrusion detectors from adversarial attacks, preserving efficacy and performance. Conversely, Zhou et al. [124] introduced HAA, a hierarchical adversarial attack strategy for GNN-based IoT detectors, which reduces classification accuracy by over 30% through minor perturbations and node prioritization techniques. Moreover, FL has gained attention for its ability to handle privacy by decentralizing data from IoT devices and aggregating the global model on a centralized server. The authors in [10] employed FL with an autoencoder for botnet detection, using edge devices to process IoT traffic and a global model to aggregate updates, achieving 99% classification accuracy. The study employed an ANN as a global model for federated intrusion detection in IoT healthcare, enhancing performance and defending against poisoning attacks [125].

**Table 7:** Summary of reviewed ML models for IoT-based malware detection: dataset sources, feature details and experimental results.

Reference	Data Source	Feature Category	Feature Name	ML Algorithms	Accuracy (%)
[68]	IoT-23 dataset	**Static**	Network capture files include IP address, ID of the capture, protocol, etc.	RF, NB, MLP, SVM, and AdaBoost	99.5
[89]	NSS Mirai Dataset latest relevant, balanced datasets https://www.stratosphereips.org/datasets-iot23 (accessed on 3 February 2025)	Static	Alert level (source and destination IP addresses, C&C activities, protocol) and packet-level features (IP address or port number, packet size, etc.)	CNN	99
[90]	ARM-based IoT	Static	Opcode features	RNN and CNN	99.98
[91]	Executable and Linkable Format (ELF) file templates are executed in the QEMU sandbox	Dynamic	System call graph	CNN	97
[102]	KISA-data challenge 2019-Malware.04, provided by the Korea Internet & Security Agency	Hybrid	Opcode and API call sequences	Bi-LSTM and spatial pyramid pooling network (SPP-Net)	92.09
[92]	Network traffic is collected from external repositories	Dynamic	2D image-based network traffic features	Neural network	91.32
[69]	Bot-IoT, MedBIoT, and MQTT-IoT-IDS2020 datasets	Dynamic	Packet-level metadata of the raw PCAP file	DT, RF, K-nearest neighbor (KNN), and extreme gradient boosting (XGB)	99.5 with RF
[93]	MedBIoT dataset [126]. IoTID (IoT network intrusion dataset) http://dx.doi.org/10.21227/q70p-q449 (accessed on 4 February 2025)	Dynamic	PCAP files	LSTM, RNN, and DT, respectively	98.71
[124]	UNSW-SOSR2019	Static	Network packets (source IP, destination IP, timestamp, traffic flows, etc.)	Graph neural network (GNN)	-
[10]	N-BaIoT dataset	Network	IoT network traffic	MLP and Auto Encoder	99%
[125]	Bot IoT dataset	Network	IoT network traffic	ANN	99%

**Summary of research trends on malware detection on IoT platform**

Table 7 summarizes IoT malware detection systems, detailing data sources, features, detection models, and accuracies.

**Dataset utilization and challenges**: Table 7 shows that datasets such as IoT-23, MedBIoT, and Bot-IoT were frequently utilized. However, issues such as dataset imbalances and limited architectural diversity remain unclear.

**Diverse feature categories**: IoT malware detection employs a mix of static (e.g., network packet features and opcode sequences) and dynamic (e.g., system call graphs and network traffic metadata) features, with some methods integrating hybrid approaches (e.g., opcode and API calls).

**Machine learning algorithms**: A wide range of ML algorithms, including RF, CNN, RNN, Bi-LSTM, and GNN, has been utilized. The RF and CNN models dominate owing to their high accuracy and adaptability to IoT-specific constraints.

**Image-based detection advances in sandboxing:** Image-based approaches and sandboxing improvements, such as QEMU-based execution and system behavior capture, have addressed limitations in resource-constrained IoT environments while enhancing the malware detection performance.

**Adversarial vulnerabilities:** Many existing models are susceptible to adversarial attacks, which reduces their real-world applicability.

### 6.4. Malware Detection on Cloud Platform

Malware detection in cloud platforms is becoming increasingly vital as organizations move data and services to the cloud. This approach leverages the cloud infrastructure to identify malicious software by monitoring activities, analyzing data, and security checks. Unlike traditional systems, cloud environments pose unique challenges due to their distributed architecture, multi-tenancy, and scalability. The dynamic and large-scale nature of the cloud enables rapid malware propagation, outpacing traditional detection methods. Detection agents on cloud servers provide security services, allowing users to upload files and receive malware reports.

Xiao et al. [127] proposed a cloud-based malware detection scheme utilizing Q-learning to optimize the offloading rate for mobile devices without prior knowledge of trace generation or radio bandwidth. They employed the Dyna architecture and post-decision state learning to enhance performance and expedite the reinforcement learning process. Testing revealed that their scheme improved detection accuracy by 40%, reduced delay by 15%, and increased mobile device utility by 47% with 100 devices, thereby enhancing overall performance. Additionally, Yadav R. Mahesh [64] introduced a malware detection system for cloud environments using a novel consolidated Weighted Fuzzy K-means clustering algorithm with an Associative Neural Network (WFCM-AANN). The proposed classifier identified malware with a high detection precision of 92.45%, surpassing existing classifiers. Similarly, Mouratidis et al. [128] introduced a security modeling language for cloud environments, integrating security requirements with cloud concepts and leveraging automated analysis to enhance security insights. Moreover, in [129], the researchers developed the CMD_2024 dataset by integrating static and dynamic features to enhance cloud malware detection. By leveraging integrated deep learning models, the study reported outstanding performance, achieving 99.42% binary and 86.97% multiclass classification accuracy.

**Summary of research trends on malware detection on cloud platform**

According to the studies reviewed in this work, advanced methods such as Q-learning and Weighted Fuzzy K-means clustering combined with neural networks have shown promising results. However, large-scale and highly variable cloud environments render malware detection challenging. Hence, existing solutions often lack scalability to handle the rapid increases in traffic and malware propagation. To overcome this issue, adaptive ML models that can efficiently handle the dynamic and multi-tenant nature of cloud systems can be developed.

### 6.5. Discussion

Based on the extensive review of the related literature discussed in Section 6.1, Section 6.2, Section 6.3 and Section 6.4, this section provides a critical analysis of malware detection methods, focusing on their effectiveness in specific scenarios and platforms, as well as their computational cost, scalability, and challenges in real-world integration. These insights highlight the strengths, limitations, and practical applicability of the surveyed methods, offering a deeper understanding of their suitability in different environments.

The effectiveness of malware detection methods varies significantly across platforms, owing to differences in computational resources, malware characteristics, and operational constraints. For instance, CNNs and LSTMs have shown high accuracy in detecting Windows malware using static features such as opcode sequences, which often struggle with zero-day malware owing to their reliance on known patterns [76,77]. In contrast, dynamic analysis provides better detection of zero-day malware by observing runtime behavior. However, dynamic analysis is resource-intensive and requires controlled environments, making it less suitable for real-time detection of end-user devices [80,112]. Hybrid approaches that combine static and dynamic features have shown promise; however, they are computationally expensive and may not scale well for large-scale deployment [82]. CNNs excel in extracting spatial patterns from malware binaries converted into images, making them highly effective for Windows environments where malware often exhibits consistent structural patterns. However, these methods are less effective in IoT environments because of the lack of large and diverse datasets and limited computational resources of IoT devices. In contrast, RF models, which are lightweight and computationally efficient, are preferred in IoT environments but may not perform as well in cloud environments. In cloud environments, behavior-based methods such as Q-learning and Weighted Fuzzy K-means clustering adapt well to the dynamic and distributed nature of cloud systems but face scalability challenges due to the rapid increase in traffic and the multi-tenant nature of these environments [127]. On Android platforms, hybrid methods that combine static and dynamic features (e.g., opcode sequences and API calls) are effective against obfuscated malware, but face scalability challenges on low-end devices owing to their high computational costs. Overall, the success of a detection method depends on its ability to balance the accuracy, computational efficiency, and adaptability to the unique characteristics of the target platform.

## 7. Challenges Associated with Platform-Specific and Cross-Platform Malware Detection

This section outlines the research challenges related to malware detection on various platforms, including Windows, Linux, macOS, Android, iOS, IoT, and cloud environments. Additionally, we discuss cross-platform challenges particularly pertinent to machine learning (ML)-based detection methods.

### 7.1. PC Platforms (Windows, Linux, and macOS)

Personal computers (PCs), including Windows, Linux, and macOS, face significant challenges in terms of malware detection. Although these platforms share commonalities, they also exhibit unique challenges that influence detection methodologies.

#### 7.1.1. Common Challenges Across PC Platforms

**Dependency on runtime libraries:** The absence of the necessary runtime libraries or loaders is a shared problem across all platforms, hindering dynamic analysis. For instance,

- Linux: Linux systems often lack the necessary ELF loaders for executing malware samples.
- Windows: Missing or incompatible DLLs can disrupt malware detection. Windows systems face similar issues as missing or incompatible Dynamic Link Libraries (DLLs).
- macOS: macOS’s sandboxing mechanisms can restrict access to runtime environments, further complicating the execution of suspicious binaries for dynamic analysis.

**Fileless malware threats:** Fileless malware that resides in memory instead of the file system challenges the traditional detection methods on all platforms.

**Cross-platform threats:** Modern malware often targets multiple platforms and exploits shared vulnerabilities. For example, the “Mirai” botnet originated as an IoT threat but has evolved to infect Linux-based systems and even Windows devices via poorly secured remote-access protocols.

**Obfuscation and polymorphism:** Malware across Windows, Linux, and macOS frequently employs obfuscation and polymorphic techniques to evade detection. For example, the ransomware family “LockBit” encrypts its code to bypass static analysis, and similar methods are used by Linux trojans to dynamically alter their signatures.

#### 7.1.2. Windows-Specific Challenges

- Widespread usage and sophisticated attacks: Windows operating systems face unique security challenges owing to their architecture and widespread usage, making them particularly vulnerable to window-specific exploits. For example, the WannaCry ransomware exclusively targets Windows systems by exploiting the EternalBlue vulnerability in the Windows SMB protocol, causing global disruptions. Additionally, Windows-specific protocols such as NTLM (NT LAN Manager) and NetBIOS have been exploited in pass-the-hash and man-in-the-middle attacks, which are not applicable to Linux or macOS systems. These vulnerabilities are often exacerbated by Windows’ reliance on legacy systems and backward compatibility requirements, leaving systems exposed to unpatched exploits [130].

#### 7.1.3. Linux-Specific Challenges

- Kernel exploits and diverse architectures: Linux operating systems face unique challenges owing to their architecture and widespread use in servers, cloud infrastructures, and IoT devices. A notable example is the Dirty COW (CVE-2016-5195) vulnerability, a Linux-specific privilege escalation flaw in the kernel memory subsystem that allows attackers to gain root access [131]. Linux-specific package managers and dependencies can also introduce vulnerabilities if not properly maintained, as seen in attacks targeting outdated software in distributions, such as Ubuntu or CentOS. These issues are compounded by the diversity of Linux distributions that can lead to inconsistent patching and security practices.

#### 7.1.4. macOS-Specific Challenges

- Exploiting native frameworks (macOS), which are known for their robust security architecture, faces unique challenges due to their closed ecosystem and reliance on native frameworks. One notable example is gatekeeper bypass vulnerability, in which attackers exploit flaws in the macOS app verification system to execute malicious software without user consent. The reliance on proprietary frameworks such as AppleScript and iCloud integration also introduces vulnerabilities, as seen in attacks that exploit scripting vulnerabilities or iCloud phishing schemes [132].
- Perception of security: macOS users often perceive their systems as inherently secure, which attackers exploit through sophisticated phishing and malvertising campaigns.

#### 7.1.5. Summary of Commonalities and Differences

Table 8 highlights key differences and commonalities in malware detection challenges across Windows, Linux, and macOS, providing insights into platform-specific vulnerabilities and security considerations and protocols.

### 7.2. Mobile Platforms (Android and iOS)

#### 7.2.1. Android-Specific Challenges

- **OS update delays:** the dependency on multiple manufacturers slows OS updates, leaving outdated devices vulnerable.
- **Third-party apps:** third-party applications increase risks to device security and user privacy.
- **Device diversity:** the variety of Android devices and OS versions complicates uniform patching and security protocols.

#### 7.2.2. iOS-Specific Challenges

- **Jailbreaking risks:** jailbreaking removes iOS restrictions, exposing devices to malware and unauthorized app installations [133].
- **iCloud phishing and account takeovers**: attackers use phishing to steal iCloud credentials, enabling data theft and device tracking.

#### 7.2.3. Summary of Mobile Platform Differences

Table 9 highlights key differences and commonalities in malware detection challenges and security features between Android and iOS, providing insights into platform-specific risks, update mechanisms, and security controls.

### 7.3. IoT-Specific Challenges

- **Device diversity:** the rapid growth and heterogeneity of IoT devices create challenges for uniform malware detection.
- **Resource constraints:** limited computational resources render IoT devices more vulnerable to malware.
- **Dataset limitations:** the lack of valid large-scale IoT malware datasets limits machine learning model training.

### 7.4. Cloud-Specific Challenges

- **Interconnected infrastructure:** the distributed nature of cloud systems increases the risk of malware attacks.
- **Shared responsibility model:** the shared responsibility model can obscure security accountability, limiting organizations’ visibility and hindering threat detection.
- **Scalability of attacks:** attackers can leverage the automation and scalability features of the cloud to launch large-scale attacks quickly.

### 7.5. Cross-Platform Challenges

- **Data heterogeneity:** differences in file formats, system call sequences, and behavioral patterns across platforms make it challenging to create generalized models.
- **Lack of unified datasets:** the absence of a standardized, diverse, and large-scale dataset encompassing samples from various platforms (Windows, macOS, Linux, Android, IoT, and cloud) complicates the effective training of detection models.
- **Model transferability:** ML models trained on one platform (e.g., Windows) may not generalize well to others (e.g., Linux or IoT) because of the differences in malware characteristics.
- **Performance scalability:** ensuring that detection techniques remain scalable and efficient when applied to resource-limited environments such as IoT and cloud systems remains a critical challenge.

These challenges emphasize the need for a multi-platform approach to malware detection that considers platform-specific constraints, while overcoming cross-platform issues.

## 8. Limitations in the Existing Literature and Future Research Directions

Based on a comprehensive review, we identified significant research gaps and limitations in the existing literature and proposed innovative future directions to enhance malware detection using machine learning (ML) techniques.

### 8.1. Limitations in the Existing Literature

**Lack of unified cross-platform detection frameworks**

Current ML-based malware detection systems are predominantly designed for single platforms (e.g., Windows, IoT, or cloud environments). This siloed approach limits their ability to detect and correlate threats across interconnected systems, such as PCs, mobile devices, IoT networks, and cloud infrastructures. Consequently, these systems struggle to address multi-vector attacks that exploit vulnerabilities across platforms, leading to fragmented and incomplete threat detection.

**High computational demands of ML models in resource-constrained environments**

The deployment of advanced machine learning (ML)-based ransomware detection techniques in resource-constrained environments, such as IoT and mobile devices, presents significant challenges. These devices often operate with limited processing power, memory, and energy resources, which restrict their ability to support computationally intensive ML models. Consequently, the practical implementation of state-of-the-art detection methods is hindered, limiting their applicability in real-world scenarios where lightweight and efficient solutions are critical.

**Limited interpretability of ML models**

The opacity of complex machine learning models remains a critical challenge in security-sensitive applications, limiting their trustworthiness and practical deployment. Although existing explainable AI (XAI) techniques, such as Shapley Additive Explanations (SHAP) and Local Interpretable Model-agnostic Explanations (LIME), have significantly enhanced model interpretability, there remains considerable scope for further research to optimize their effectiveness in real-time threat mitigation and decision-making scenarios.

**Limitations of large language models (LLMs) in malware detection**

Although large language models (LLMs) show promise in malware detection, several limitations hinder their adoption, including high computational costs, lack of domain-specific training data, susceptibility to adversarial attacks, scalability challenges, and ethical concerns. For example, fine-tuning LLMs (ChatGPT, Google Bart, GPT, BigBird) for malware detection often requires high-performance GPUs or TPUs [134], which are not feasible for resource-constrained environments such as IoT devices or low-end mobile devices.

### 8.2. Future Research Directions

**Development of unified cross-platform frameworks**

Future studies should focus on developing unified cross-platform malware detection frameworks that can leverage transfer and federated learning. Transfer learning can enable models to generalize knowledge from one platform to another, thereby reducing the need for extensive retraining and improving adaptability [104]. Federated learning can facilitate collaborative model training across distributed platforms without sharing sensitive data, thereby ensuring privacy and scalability [108]. In addition, researchers should explore cross-platform feature representation techniques to harmonize data from diverse environments (e.g., API calls, network traffic, and system logs) into a unified feature space. For instance, Chaganti et al. (2022) demonstrated the effectiveness of multiview feature fusion for malware classification, which can be extended to integrate features from Windows, IoT, and cloud environments, enabling more robust and generalizable malware detection [81].

**Lightweight solutions for resource-constrained environments**

To address the limitations posed by resource-constrained environments, future research should focus on developing lightweight and energy-efficient ML algorithms tailored for IoT and mobile devices. Advanced techniques, such as model pruning, quantization, and knowledge distillation, offer promising avenues for optimizing ML models without significantly compromising the detection accuracy. The technique of “model pruning” involves removing weights or parameters from a trained machine learning model to reduce its size and complexity while attempting to maintain accuracy [135]. Quantization reduces the precision of the model parameters (e.g., converting 32-bit floating-point values to 8-bit integers), which decreases the model size and accelerates the inference speed, making it more suitable for real-time applications on low-power devices. Although quantization may result in a slight loss of precision, the trade-off is often justified by efficiency gains [136]. Knowledge distillation is a method in which a large, complex model transfers its learned knowledge to a smaller, simpler model to improve the efficiency while retaining high performance. This is beneficial for creating lightweight malware detection models [137]. By leveraging these techniques, future research can bridge the gap between advanced ML-based ransomware detection and the practical constraints of the IoT and mobile environments.

**Explainable AI for enhanced transparency**

Future research should advance the explainability of ML-based cybersecurity solutions beyond post hoc methods such as SHAP and LIME by developing adaptive explainability frameworks that provide real-time dynamic interpretations during cyberattacks, enabling faster incident responses [138]. Given the heterogeneity of computing environments, multimodal explainability approaches are essential for addressing platform-specific nuances such as API call sequences for Windows malware, device interactions for IoT, and virtual machine behaviors for cloud systems [139]. Additionally, context-aware XAI models should deliver actionable insights tailored to specific incidents, while ensuring cross-platform consistency to support unified detection strategies [140]. For example, studies have shown that unified XAI frameworks can improve the interpretability of malware detection across diverse environments such as endpoints, networks, and cloud systems by maintaining consistent feature attributions and explanation formats [141]. However, achieving these goals requires addressing key challenges, including the computational overhead and integration of domain-specific knowledge.

Enhancing LLMs for scalable and secure malware detection

Future research should focus on developing lightweight LLMs (e.g., distilled or quantized models) [142] that can operate efficiently on resource-constrained devices, such as IoT sensors or mobile phones. In addition, fine-tuning LLMs on domain-specific datasets, enhancing adversarial robustness, improving interpretability through explainable AI (XAI), designing scalable deployment architectures, and ensuring ethical and privacy-preserving implementations are critical areas for future research. Moreover, integrating LLMs with neural network models can provide a more comprehensive approach for malware detection by leveraging the strengths of both techniques.

## 9. Conclusions

This study provides a comprehensive review of machine learning (ML)-based malware detection techniques across diverse platforms, each presenting platform-specific characteristics, research trends, and unique security challenges owing to distinct vulnerabilities, operational constraints, and resource limitations. Our findings reveal that although significant progress has been made in malware detection using ML models, existing solutions remain largely platform-centric, limiting their effectiveness in addressing cross-platform threats. Furthermore, issues such as data scarcity, adversarial evasion techniques, and the need for interpretable AI models pose challenges to real-world deployment. By synthesizing recent research since 2017, this study underscores the critical need for the development of adaptable, cross-platform malware detection mechanisms to combat evolving malware threats. It also highlights the importance of adopting advanced ML techniques, such as neural networks (CNNs and RNNs), privacy-preserving federated learning, LLMs, and XAI, to improve detection accuracy and interpretability. Lightweight models tailored to resource-constrained IoT and edge devices are also essential for effective deployment across increasingly interconnected ecosystems. As cybercriminals exploit vulnerabilities in interconnected systems, this study serves as a critical resource for advancing robust, interpretable, and cross-platform malware detection systems to address the evolving cybersecurity landscape.

## Figures and Tables

**Table 2:** Categorization of platform-specific features: static, dynamic, and memory-based approaches across file formats and operating environments.

Platform	File Format	Static Features	Dynamic Features	Memory Features
**Windows**	Executable (EXE) files.	**PE header information:** Import/export address tables, section headers, entry point address, date timestamp, code section size. **File metadata**: Size, creation/modification dates, access permissions. **Strings:** IP addresses, domain names. **Opcode sequences**: An opcode is an instruction executed by a CPU, describing an executable file’s behavior. Hence, opcode sequences are the specific sequences of operations extracted from the binary code.	**API calls**: Sequence and types of Windows API calls (e.g., CreateProcess, WriteFile). **Registry modifications:** Registry key creation, deletion, or modification. **File system modifications:** Deletes, creates, or overwrites the existing file, encrypts all or a subset of files in case of ransomware. **Host logs:** Events extracted from host logs. **Network activity:** Source and destination IP addresses, TCP ports, Domain Names System (DNS) requests, and network protocols (e.g., HTTP, HTTPS, SMTP, etc.). **Resource usage:** Higher CPU or memory usage may indicate the presence of malware in the system.	Windows memory dumps.
**Linux**	Executable and Linkable Format (ELF): code, data, and metadata for execution.	**ELF header information:** Malware developers manipulate ELF headers to evade or crash standard analysis tools [46]. **Internal libraries:** Most Linux malware is statically linked to its libraries, eliminating external dependencies [46]. **Shared libraries**: List of dynamically loaded libraries. **Sections and segments**: Information on the .text (code) and .data (global variables) segments.	**System call patterns**: Frequency and type of system calls. **Network behavior**: Monitoring outgoing/incoming connections and socket creation.	**Sections and segments**: Memory segments (.text, .data, .bss).
**macOS**	**Mach-O files:** native executable format for macOS.	**Code signatures**: Presence and structure of code signing. **Dynamic libraries**: Information on loaded libraries (DYLIBs).	**File activity:** Monitor file creations, deletions, modifications, and access patterns. **Inbound and outbound traffic:** Observe and analyze all network traffic, including DNS, HTTP requests, and other communication protocols. **Service start/stop:** Track each modification linked to service operation. **TI reputation services:** Utilize threat intelligence to detect malicious files, IP addresses, and domains.	**Sandboxing**: Memory protection through entitlements.
**Android**	**APK (Android Package Kit) files:** It is a compressed archive that includes all the resources needed to distribute and install applications on Android devices.	**Strings:** Domain names, IP addresses, and ransom notes in case of ransomware attack. **Permissions analysis**: The set of permissions requested by the app to the users (e.g., camera access, network communication, Bluetooth, contacts, and more). **Manifest information**: Details about application components (e.g., activities, services, and receivers). **Intents:** Allows communication between various components of an app. **API calls:** API calls enable inter-application communication to detect malicious behavior.	**Behavioral features**: Network communication, SMS, data storage behavior. **File system features:** Similarly to PCs, features extracted from a mobile device’s file system can indicate the presence of malware. **User interaction:** Detecting ransomware can be achieved by correlating user interactions with application runtime events [47]. **System resource analysis:** CPU, memory and battery, process reports and network usage. **Network traffic analysis:** URLs, IPs, network protocols, certificates, non-encrypted data.	**Embedded files**: Presence of assets (e.g., shared libraries) impacting memory allocation. **Memory dumps:** A snapshot of Android’s memory that captures all data and processes in the RAM at a specific time, including system processes, application data, and temporary data from various programs.
**iOS**	iOS App Store Package (IPA): specific to iOS for app distribution.	**Code signing:** Verification of signatures. **Sandboxing and entitlements: Permission restrictions.**	**Objective-C method calls**: Runtime behavior. **Dynamic behavior**: API usage patterns (e.g., contacts, location access). **Data encryption**: Encrypted data usage.	**Entitlements**: Defines memory boundaries through sandboxing.
**IoT**	Various formats (e.g., BIN, HEX, Linux executables).	**Firmware version**: Metadata, updates, and patches. **Opcode sequences:** Extracting operational codes after disassembling the binary file. **Control-flow graph (CFG):** Extracting from the assembly file. **API calls**: Extracting from the binary.	**Network traffic**: Service type (http, smtp, ftp, etc.), device communication protocols (e.g., MQTT, CoAP), packet size transmitted by source IP address, etc. **Device-specific behavior**: Interactions with sensors, actuators, device ports. **System calls:** Timestamp, return value, arguments, and name of each system call. **Resource utilization-based features: 'CPU usage, process usage, and RAM usage**.	**System call sequences**: System-level commands specific to device memory. **Memory-mapped IO**: Monitoring interactions with memory-mapped I/O (MMIO). **Memory buffer usage**: Analysis of memory buffers for potential overflows.
**Cloud**	VM disk images (e.g., VMDK, QCOW2), container formats (e.g., Docker images).	**VM metadata**: Hypervisor information (e.g., VM details). **Data storage patterns**: Interactions with cloud storage. **Strings and n-grams**	**API usage patterns**: Cloud-specific API calls (AWS SDK, Google Cloud API). **Container activity**: Monitoring processes and network activity in containers. **System calls:** Extracted from the interactions between applications and the OS’s kernel during runtime.	**Virtual memory dumps:** Contains memory-specific features (system calls, memory access).

**Table 3:** Summary of machine learning algorithms applied in various studies across diverse platforms for malware detection.

ML Techniques	Algorithms	References
**Traditional machine learning algorithms**		
**Support Vector Machines (SVMs):** This method employs a hyperplane to maximize the margin between malicious and benign samples, proving effective for high-dimensional data.	SVM	[66,67,68]
**K-Nearest Neighbors (KNN):** This algorithm classifies samples based on the predominant class of their nearest neighbors, utilizing feature similarity as the primary criterion.	KNN	[55,69]
**Logistic Regression (LR):** This approach classifies malware by modeling the relationship between features and binary outcomes (malicious or benign) utilizing a sigmoid function. The sigmoid function converts input values to a range of 0 to 1, making it ideal for interpreting results as probabilities. It is used for binary classification tasks, especially in logistic regression and neural networks.	LR	[70,71,72]
**Naïve Bayes (NB):** A probabilistic approach that assumes feature independence, which is efficient for text-based malware detection.	NB	[67,68]
**Decision Trees (DTs):** Decision trees are a supervised learning method that classify data by building a tree-like model. The process identifies the most critical features and splits the data into subsets based on these attributes to form nodes. It recursively classifies each node until a final decision is reached as benign or malware.	DT	[69,70]
**Ensemble learning algorithms**		
**Random Forest (RF):** This approach constructs multiple decision trees and aggregates their outputs through majority voting or averaging, thereby enhancing robustness and accuracy.	RF	[67,68,69,70,72,73,74]
**Gradient Boosting (e.g., XGBoost, LightGBM):** This approach sequentially constructs weak learners, specifically decision trees, to minimize errors, thereby providing high accuracy in the analysis of structured malware data.	Gradient Boosting	[72]
	XGBoost	[69,72]
**AdaBoost:** This approach focuses on challenging samples by modifying weights during the training process, thereby combining weak classifiers into a robust one.	AdaBoost	[68,74]
**Bagging:** The Bagging technique randomly divides the dataset into multiple subsets (bootstraps) based on instances, each with unique instances, and then aggregates the results from models trained on these subsets to enhance generalization.		
**Deep learning algorithms**		
**Convolutional Neural Networks (CNNs):** This approach demonstrates efficacy in image-based malware detection, utilizing automated extraction of spatial features from transformed malware binaries.	CNN	[70,75,76,77,78,79,80,81,82,83,84,85,86,87,88,89,90,91]
**Recurrent Neural Networks (RNNs):** This method facilitates the analysis of sequential data, including API call sequences and opcode patterns, for behavioral-based malware identification.	RNN	[75,90,92,93]
**Long Short-Term Memory (LSTM):** A variant of the recurrent neural network (RNN) that effectively captures long-term dependencies, particularly applicable for time-series analysis of dynamic malware features.	LSTM	[76,80,82,87,93,94,95,96,97,98,99,100,101,102]
**Gated Recurrent Unit (GRU):** It is a type of recurrent neural network (RNN) designed to process sequential data, such as time series or text. This model is more computationally efficient than LSTMs due to fewer parameters and the absence of a separate output gate.	GRU	[95]
**Generative Adversarial Networks (GANs):** This process generates synthetic malware samples for data augmentation, thereby enhancing the efficacy of detection systems with limited datasets.	GAN	[84]
**Autoencoders:** Autoencoders are unsupervised neural networks used for dimensionality reduction, feature extraction, and anomaly detection. They aim to learn a compressed representation of the input data (encoding) and then reconstruct the input (decoding) as accurately as possible.	VAEs, Sparse Autoencoders etc.	[103]
**Transformer Models (e.g., BERT):** Transformers are advanced deep learning architectures based on attention mechanisms designed to handle sequential or contextual data effectively.	BERT	
**Transfer Learning (TL):** This is a deep learning approach where a model pre-trained on one task or dataset is reused and fine-tuned for a related but different task. It is particularly effective when the target dataset is small or lacks diversity.	Pre-trained CNNs like Inception, VGG, ResNet50, etc.	[104,105,106]
**Multilayer Perceptron (MLP):** It is a type of artificial neural network (ANN) consisting of multiple layers of nodes. It is commonly used in supervised learning tasks such as classification and regression.	MLP	[55,68,70,73,85,107]
**Federated Learning (FL):** FL is an emerging AI model in which ML models are trained locally on edge devices such as smartphones and IoT devices, without sharing raw data. Instead, only model parameters and gradients are exchanged with a global model, preserving user privacy, and enhancing security. However, its effectiveness depends on device capabilities and communication overhead.	-	[10,108]
**Large Language Models (LLMs):** The ability of LLMs to capture contextual relationships enables the identification of subtle patterns indicative of malicious activities. LLMs assist in automating threat analysis, improving detection accuracy, and aiding in malware classification.	GPT, BERT, ChatGPT-4, Claude	[8,9]

**Table 4:** Summary of reviewed ML models for Windows-based malware detection: dataset sources, feature details, experimental results, and limitations.

Reference	Data Source	Feature Category	Feature Name	ML Algorithms	Result (Accuracy)	Limitations
**Static feature-based malware detection**						
[75]	Malimg	Static	Opcode sequences	Deep RNN	96%	It requires significant computational resources.
[76]	Microsoft BIG 2015	Static	Opcodes, images, byte sequence, etc.	DNN, LSTM, and CNN.	98.35%	It is useless against zero-day malware.
[104]	BIG 2015, Malimg, MaleVis and Malicia dataset	Static	2D images	DenseNet	98.23%	It has high false negatives and highly imbalanced datasets.
[77]	Microsoft BIG 2015	Static	Image-based opcode features	CNN	99.12%	Outdated dataset.
[105]	Malimg dataset, Microsoft BIG 2015	Static	Grayscale images from PE files	VGG16, VGG19, ResNet50, and inceptionV3	98.92%	Cannot detect malware packed using advanced techniques.
[109]	Malimg	Static	Static signatures	ATT-DNNs	98.09%	Cannot detect obfuscated malware.
[78]	Malware API-class	Static	Executable file to static images	CNN	98.00%	_
[79]	VirusShare, Hybrid Analysis	Static	Executable file to static images	Xception Convolutional Neural Network (CNN)	98.20%	_
[110]	Microsoft BIG 2015	Static	Malware binary files into static images	DNN	97.80%	_
**Dynamic feature-based malware detection**						
[94]	VirusShare	Dynamic	Sequences of API calls	Bi-LSTM	97.31%	Limited to executing samples in a Windows 7 environment.
[111]	Custom datasets	Dynamic	Sequences of API calls	Markov chain representation	99.7%	-
[112]	VirusTotal	Dynamic	API calls	LSTM	95%	Limited to executing samples in a Windows 7 environment.
[95]	VirusTotal	Dynamic	API call sequences	LSTM and GRU	96.8%	Highly imbalanced dataset.
[66]	CA Tech- neologises VET Zoo	Dynamic	Runtime behavior	MRed, ReliefF, SVM	99.499%	High computational complexity.
[96]	Audit log events	Dynamic	Process names, action types, and accessed file	LSTM	91.05%	High false positives and lack of scalability.
[80]	Multiclass dataset (Ember Dataset, private dataset)	Dynamic	Loaded DLLs, registry changes, API call sequences and file changes	CNN-LSTM	96.8%	Susceptible to adversarial attacks.
**Hybrid feature-based malware detection techniques**						
[81]	VirusTotal	Hybrid (static and dynamic)	Combination of static and dynamic features (PE section, PE import, PE API, and PE images)	CNN	97%	Failed to validate the robustness against adversarial attacks.
[73]	The Korea Internet & Security Agency (KISA)	Hybrid	Size of file and header, counts of file sections. Entropy, file system changes API call, DLL-loaded info, network activities, etc.	RF, MLP	85.1%	Extensive time is needed for feature extraction.
[106]	VirusShare	Hybrid	Image-based static and dynamic features	VGG16	94.70%	-
[113]	VirusShare	Hybrid	Function length frequency representation, registry activities, API calls, and file operation features	SVM	97.10%	Small dataset.
[82]	VirusTotal	Hybrid	Opcodes and system calls	CNN, LSTM, and an attention-based LSTM	99%	High computational cost.
**Memory feature-based malware detection techniques**						
[83]	Dumpware10	Memory	Memory images of running processes	CNN	98%	Malware processing cost is high under limited resource capabilities.
[84]	Dumpware10, BIG2015 dataset	Memory	Memory images of running processes	GAN and CNN	99.86% for BIG2015 dataset	Only one type of data, like bytes, is used. Need to make the dataset more diverse.
[85]	CIC-MalMem-2022 https://www.unb.ca/cic/datasets/malmem-2022.html (accessed on 3 February 2025)	Memory	Memory images of running processes	CNN and MLP	99.8%	Training time complexity and vulnerability to adversarial attacks.
[70]	CIC-MalMem-2022 https://www.unb.ca/cic/datasets/malmem-2022.html (accessed on 3 February 2025)	Memory	Multi-memory features	RF, DT, LR, MLP, and CNN	99.89%	-

**Table 5:** Summary of reviewed ML models for Linux-based malware detection: dataset sources, feature details, and experimental results.

Reference	Data Source	Feature Category	Feature Name	ML Algorithms	Result (Accuracy)
[107]	AndroZoo, VirusShare, and clean Ubuntu libraries	Static	Assembly instructions (control-flow graphs)	MLP	96.82%
[115]	VirusShare	Static	Strings from binary data	DNN	94%
[74]	VX heavens	Dynamic	System calls	J48, random forest, AdaBoostM1 (J48), and IBk	98%
[86]	VirusShare	Memory	Memory dumps	CNN	99.9%
[46]	VirusTotal and ViruShare	Memory	Multi-memory features	DNNs	98.8%

**Table 6:** Summary of reviewed ML models for Android-based malware detection: dataset sources, feature details, experimental results, and limitations.

Reference	Data Source	Feature Category	Feature Name	ML Algorithms	Accuracy	Limitations
**Static feature-based Android malware detection techniques**						
[118]	MalGenome	Static	Call graphs	GCN	98.99%	Lack of representative of real-world scenarios.
[87]	Contagio Mobile	Static	Opcode sequences	CNN-LSTM	91.42%	Unable to manage obfuscated malware.
[71]	MalDroid-2020 dataset	Static	Opcode sequences (histograms of n-grams)	LR	93.56%	Adversarial attack resistance and handling evolving malware are not addressed.
[97]	CIC-Inves2017	Static	Opcode sequences	LSTM	96%	Small dataset (1500 apps).
[72]	Drebin, VirusShare, AndroZoo	Static	Permissions, intents	Base models (LR, MLP, and SGD), ensemble learning	99.1%	-
[88]	Drebin dataset	Static	Opcode sequences, permissions, API calls	CNN	99.92%	Lack of malware diversity and scalability.
**Dynamic feature-based Android malware detection techniques**						
[67]	McAfee	Dynamic	Actions/events	Base models (NB, SL, SVM RBF, J48, PART, RF), deep learning	97.8%	-
[98]	Google Play Store	Dynamic	API calls	Bi-LSTM	97.22%	High detection time.
[119]	Drebin dataset	Dynamic	Network traffic Permissions, intents, API calls	C4.5	97.89%	Small dataset.
[99]	MalGenome	Dynamic	System call sequences	LSTM	99.23%.	-
[100]	Custom dataset	Dynamic	API and system call sequences	LSTM	96.8%	
**Hybrid feature-based Android malware detection techniques**						
[67]	McAfee	Hybrid	Permissions, intents, API calls, actions/events	Base models (NB, SL, SVM RBF, J48, PART, RF), deep learning	99.6% detection	-
[55]	Contagio Mobile, https://contagiominidump.blogspot.com/ (accessed on 3 February 2025) VirusShare and Genome	Hybrid	Runtime behaviors across various levels—kernel, application, user, and package	K-NN, LDC, QDC, MLP, Parzen Classifier (PARZC) and RBF	96%	This method is susceptible to mimicry attacks and ineffective against unknown malware.
[101]	VirusShare, Drebin, DroidAnalytics and CICInvesAndMal2019/2000 https://www.unb.ca/cic/ (accessed on 3 February 2025)	Hybrid	Permission requests, API and system call sequences, opcode sequences, and graph structures, including abstract syntax trees, control-flow, and data-flow graphs	Bi-LSTM and GNN	95.94%	Need more scalable static analyses.
[103]	CICMal-Droid2020	Hybrid	Permissions, intents, system calls, composite behaviors, and network traffic packets	Pseudo-label stacked autoencoder (PLSAE)	98.28%	-
**Memory feature-based Android malware detection techniques.**						
[85]	AndroZoo project https://androzoo.uni.lu/ (accessed on 3 February 2025)	Memory	Process memory dumps	Ensemble of MLP and CNN	94.3%	Vulnerable to adversarial attacks.

**Table 8:** Comparison of commonalities and differences in malware detection challenges across Windows, Linux, and macOS.

Aspect	Windows	Linux	macOS
**Runtime issues**	DLL dependencies	ELF loader challenges	Sandboxing restricts runtime access
**Cross-platform threats**	Vulnerable to malware like Mirai	Targeted by cross-platform threats	Exploits shared vulnerabilities
**Unique vulnerabilities**	EternalBlue (WannaCry) exploits SMB protocol	Dirty COW (CVE-2016-5195) targets kernel	Gatekeeper bypass allows malicious execution
**Perception of security**	High attack surface due to widespread usage	Perceived as secure but targeted for IoT/cloud	Perceived as secure, leading to lax practices
**Protocols and frameworks**	NTLM and NetBIOS exploited	Kernel vulnerabilities targeted	AppleScript and iCloud exploited

**Table 9:** Comparison of malware detection challenges and security features between Android and iOS.

Aspect	Android	iOS
**OS update delays**	Delays due to dependency on manufacturers and carriers, leaving devices vulnerable.	Timely updates controlled by Apple, ensuring uniform distribution.
**Third-party apps**	Third-party app stores and sideloading increase security risks.	Apps restricted to App Store with strict review guidelines, reducing risks.
**Device diversity**	Wide range of devices and custom OS versions complicate uniform patching.	Limited device variants and centralized control ensure consistent security.
**Jailbreaking risks**	Not applicable (rooting exists but is less common).	Jailbreaking removes iOS restrictions, exposing devices to malware.
**iCloud phishing**	Not applicable (Google account phishing exists but is less targeted).	iCloud phishing leads to account takeovers and data theft.
